# IMPACT OF STRUCTURAL RACISM ON COGNITIVE DECLINE

**DOI:** 10.1093/geroni/igae098.0622

**Published:** 2024-12-31

**Authors:** Talha Ali, Joshua Cohen, Owais Gilani, Belinda Needham, Darya Dokshina

**Affiliations:** Tufts University, Medford, Massachusetts, United States; Massachusetts General Hospital, Boston, Massachusetts, United States; Tufts University School of Medicine, Boston, Massachusetts, United States; University of Michigan, Ann Arbor, Michigan, United States; University of Michigan, Ann Arbor, Michigan, United States

## Abstract

Black individuals are twice as likely as White individuals to develop cognitive impairment. However, little empirical research has investigated the sources underlying these racial disparities. We examine the effects of exposure to structural racism as a fundamental cause of racial disparities in the risk of subjective cognitive decline among older adults. Data on subjective cognitive decline from the 2015-2016 Behavioral Risk Factor Surveillance System (BRFSS) were analyzed among adults aged 45 years and older. We operationalized structural racism as an index of the number of racist vs. neutral vs. anti-discriminatory state-level policies. BRFSS participants were assigned structural racism exposure based on their state of residence at the time of the survey. The association between exposure to structural racism and the risk of subjective cognitive decline was examined using multilevel logistic regression models. Interaction terms were used to examine how this association was modified by individual-level race. American Indian Alaska Native, multiracial, and Hispanic individuals had the highest reports of experiencing subjective cognitive decline. Black individuals were the most likely to live in states with high levels of structural racism. Greater exposure to structural racism was associated with higher odds of reporting subjective cognitive decline. This association was modified by individual-level race and ethnicity, such that the risk of subjective cognitive decline decreased with increasing exposure to structural racism among Hispanic individuals. These findings warrant further research into exploring the social and emotional factors that offer resilience among Hispanic older adults.

